# Realizing Ultrahigh Near-Room-Temperature Thermoelectric Figure of Merit for N-Type Mg_3_(Sb,Bi)_2_ through Grain Boundary Complexion Engineering with Niobium

**DOI:** 10.1021/acsami.4c12046

**Published:** 2024-09-24

**Authors:** Melis Ozen, Arda Baran Burcak, Duncan Zavanelli, Minsu Heo, Mujde Yahyaoglu, Yahya Oz, Ulrich Burkhardt, Hyun-Sik Kim, G. Jeffrey Snyder, Umut Aydemir

**Affiliations:** † Graduate School of Sciences and Engineering, Koç University, Istanbul 34450, Türkiye; ‡ Boron and Advanced Materials Application and Research Center, Koç University, Istanbul 34450, Türkiye; § Turkish Aerospace, R&D and Technology Directorate, Ankara 06980, Türkiye; ∥ Department of Chemistry, Koç University, Sariyer, Istanbul 34450, Türkiye; ⊥ Department of Materials Science and Engineering, Northwestern University, Evanston IL-60208, United States; # Department of Materials Science and Engineering, University of Seoul, Seoul 02504, South Korea; ∇ Max-Planck-Institut für Chemische Physik fester Stoffe, Dresden 01187, Germany

**Keywords:** thermoelectric, Mg_3_(Sb, Bi)_2_, niobium, grain boundary complexion, two-phase model

## Abstract

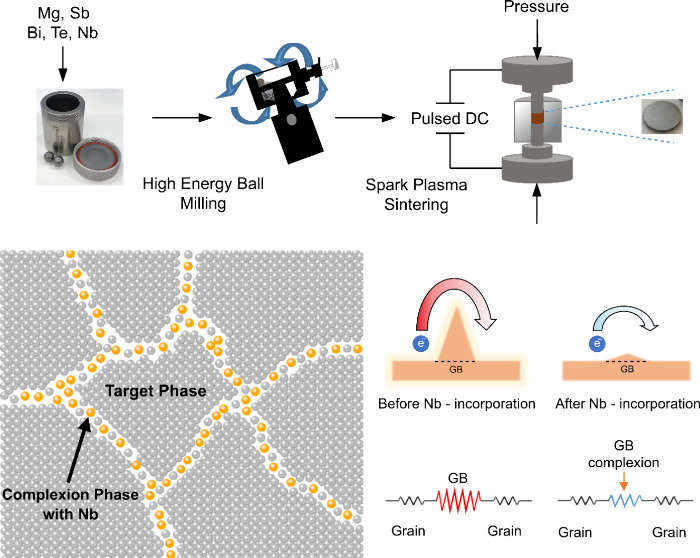

Despite decades of extensive research on thermoelectric materials, Bi_2_Te_3_ alloys have dominated room-temperature applications. However, recent advancements have highlighted the potential of alternative candidates, notably Mg_3_Sb_2_–Mg_3_Bi_2_ alloys, for low- to mid-temperature ranges. This study optimizes the low-temperature composition of this alloy system through Nb addition (Mg_3.2–*x*_Nb_*x*_(Sb_0.3_Bi_0.7_)_1.996_Te_0.004_), characterizing composition, microstructure, and transport properties. A high Mg_3_Bi_2_ content improves the band structure by increasing weighted mobility while enhancing the microstructure. Crucially, it suppresses detrimental grain boundary scattering effects for room-temperature applications. While grain boundary scattering suppression is typically achieved through grain growth, our study reveals that Nb addition significantly reduces grain boundary resistance without increasing grain size. This phenomenon is attributed to a grain boundary complexion transition, where Nb addition transforms the highly resistive Mg_3_Bi_2_-rich boundary complexion into a less resistive, metal-like interfacial phase. This marks the rare demonstration of chemistry noticeably affecting grain boundary interfacial electrical resistance in Mg_3_Sb_2_–Mg_3_Bi_2_. The results culminate in a remarkable advancement in *zT*, reaching 1.14 at 330 K. The device *ZT* is found to be 1.03 at 350 K, which further increases to 1.24 at 523 K and reaches a theoretical maximum device efficiency (η_max_) of 10.5% at 623 K, underscoring its competitive performance. These findings showcase the outstanding low-temperature performance of *n*-type Mg_3_Bi_2_–Mg_3_Sb_2_ alloys, rivaling Bi_2_Te_3_, and emphasize the critical need for continued exploration of complexion phase engineering to advance thermoelectric materials further.

## 1 Introduction

Concerns on environmental pollution, global warming, and greenhouse gas emissions have been increasing over the last decades. Therefore, many countries have agreed on the net zero emission, climate policy and integration of green energy systems by 2050.^1^ Considering more than half of the energy is lost as waste heat by industry, transportation, or everyday activities, harvesting waste heat for alternative energy presents a compelling solution, significantly mitigating greenhouse gas emissions and curbing overall energy consumption with notable efficiency. The thermoelectric effect relies on converting the temperature gradient effectively to electricity and vice versa. Compared to high-quality waste heat (above 573 K), recovering low-grade waste heat, which makes up to ca. 90% of the source, is challenging due to its lower energy density.^2−4^ So far, the thermoelectric cooling market has been essentially dominated by Bi_2_Te_3_-based materials.^5^ However, the diminishing availability and toxicity of the Te element are reducing the practicality of Bi_2_Te_3_, prompting a shift in focus toward alternative materials.^6^ While the majority of thermoelectric studies have concentrated on materials with high thermoelectric figure-of-merit, *zT* values (*zT* = *S*^2^σ*T*/κ, where *S* is the Seebeck coefficient, σ is electrical conductivity, *T* is the absolute temperature, and κ is thermal conductivity) in mid- and high-temperature ranges, such as lead chalcogenides,^7−9^ skutterudites,^10−13^ and half-Heuslers,^14−16^ there has been a recent surge in interest in Mg_3_Sb_2_ and Mg_3_Bi_2_-based materials. These alternatives are gaining attention for both low and mid-temperature applications, providing a potential substitute for Bi_2_Te_3_ materials. Indeed, recent studies have showcased impressive thermoelectric performances achieved through Mg_3_Sb_2_ and Mg_3_Bi_2_-based thermoelectric materials and modules.^2−4,17−22^

Mg_3_Sb_2_ is known as a great candidate for thermoelectric applications due to the characteristic properties obeying the phonon-glass electron-crystal concept, displaying inherently low κ along with favorable electronic transport behavior.^23−25^ To date, various strategies have been applied to enhance the thermoelectric efficiency of the Mg_3_Bi_2_ system, such as microstructure engineering, carrier concentration optimization, band structure modification, annealing, etc.^4,19,21,25−33^ Among these studies, alloying with Mg_3_Bi_2_ has notably enhanced the *zT* which has been attributed to an improved band structure (higher weighted mobility) and modifying scattering mechanisms.^4,19,22,34−39^

Despite their potential, the room temperature thermoelectric properties of Mg_3_Sb_2_- and Mg_3_Bi_2_-based thermoelectric materials are known to be severely limited due to grain boundary electrical resistance. Originally thought to be due to ionized impurity scattering^36^ that could be modified by incorporation of other elements such as Nb; it was discovered that a thermally activated grain boundary electrical resistance leads to low σ and, therefore, low *zT* at room temperature.^31,40,41^ Luo et al.^33^ through experimental techniques including nuclear magnetic resonance (NMR), atom probe tomography (APT), and scanning/transmission electron microscopy (SEM/TEM), along with theoretical work by Gorai et al.^42^ demonstrated that Nb does not significantly diffuse into the Mg_3_Sb_2_ crystal structure and thus could not alter the doping or scattering in Mg_3_Sb_2_ but instead was clearly present in and near the grain boundaries where it could enhance the grain growth and therefore diminish the impact of grain boundaries. Thus, it could be assumed that Nb and other transition metal impurities such as Cu, Ta, Co, Mn, and Hf function primarily to increase grain size.^3,30,31,36−38,43,44^ The hopping-like conductivity at the grain boundaries leads to a dramatic reduction in grain boundary electrical resistance at higher temperatures, allowing decent thermoelectric performance at high temperatures in *n*-type Mg_3_Sb_2_. However, for good room temperature performance, the grain boundary resistance must be reduced, usually by growing large grains.^29,31,45^

These results lead to a new strategy to suppress grain boundary resistance by introducing a wetting phase that aids in grain growth beyond heat treatments such as annealing and adjusting sintering temperatures.^27,46,47^ Additionally, samples with a greater amount of Mg_3_Bi_2_ possess larger grain sizes and show lower grain boundary electrical resistances.^4^ Liu et al. have achieved a *zT* of ∼1.0 at 425 K for Mg_3.2_ Sb_1.5_ Bi_0.49_ Te_0.01_ Cu_0.01_ by utilizing a Mg_2_Cu wetting phase introduced during the sintering process. The Cu addition was thought to maximize the Mg stoichiometry along the grain boundaries, thereby reducing the grain boundary resistance.^3,17^

Recent studies confirm a weak doping effect of Nb metal for Mg_3_(Sb,Bi)_2_ systems, consistent with the concept of wetting grain boundaries.^36,48,49^ A recent study revealed a maximum *zT* of 2.04 at 798 K for Mg_3.1_Nb_0.1_Sb_1.5_Bi_0.49_Te_0.01_,^50^ which shows great potential for mid-temperature applications. Another study on the addition of Nb to Mg_3_Sb_2_ with a higher proportion of Mg_3_Bi_2_ achieved a room temperature *zT* of 1.02.^51^ This improvement is attributed to Nb’s optimization mechanisms within the grains and at the grain boundaries, which prevent Mg vacancy accumulation and facilitate electron transmission both within and across grains.

**Figure 1:**
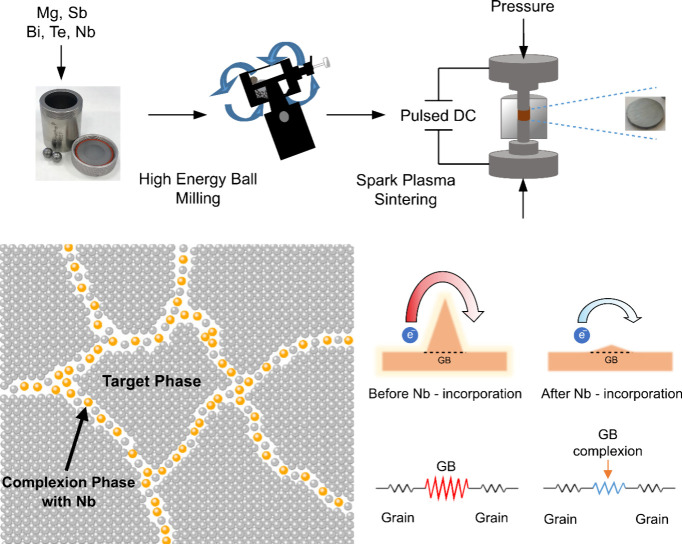
Visualization of the improved electronic properties through grain boundary engineering.

An alternative strategy to tuning the grain size of *n*-type Mg_3_Sb_2_-based materials is modifying the chemical composition of grain boundaries. This has been observed in other thermoelectric materials, particularly in half-Heusler alloys. In Ti-doped NbFeSb, increasing Ti content was found to lower grain boundary resistance through the formation of a Ti-rich phase at the boundaries.^52^ Similarly, in NbCoSn, Pt dopant segregation reduced scattering from grain boundaries.^53^ In all such cases, either dopant segregation or secondary phase formation along the boundaries serves to change the energy of the electronic states at the boundary. The effect of the distribution of low-energy grain boundaries on physical properties can be significant, as seen in this study. The structure and chemistry of grain boundaries depend on the type and orientation of the neighboring grains but also on the thermodynamic conditions such as temperature and chemical potential. Thus, grain boundary phases are often called “complexions” to distinguish them from bulk 3D phases (Figure 1). Complexions are essentially 2D phases that exist between 3D grains. Complexions have an energy, defined by the Gibbs excess surface energy and an excess composition (composition differs from neighboring grains) so that the complexion can be engineered with temperature and composition even without grain growth or grain reorientation. In some thermoelectric materials such as *n*-type Mg_3_Sb_2_ the complexions are known to be semiconducting with high interfacial resistance due to Mg deficiency.^54^ Reducing the impact by growing grains (or single crystals with only low-angle grain boundaries) has been successful in greatly improving thermoelectric performance at low temperatures.^3,29−31^ In principle, the properties of complexion phases should also depend on chemistry, but this has not been conclusively demonstrated in Mg_3_(Sb,Bi)_2_ until now.

As discussed above, the doping studies previously applied in this system attribute the improvement in *zT* to factors such as (i) ionized impurity scattering predominating at low temperatures, (ii) substituting Mg to tune carrier concentration, and/or (iii) reducing lattice thermal conductivity due to large atomic mass fluctuations (alloy scattering). However, recent reports using NMR, X-ray absorption spectroscopy (XAS), SEM/TEM, APT have clearly shown no evidence of metal incorporation that would alter the scattering mechanisms in this system.^55^ Moreover, single crystal studies indicate that ionized impurity scattering does not occur in Mg_3_Sb_2_. Instead, the observed increase in electrical conductivity with temperature, seen only in polycrystalline samples, is due to an additional grain boundary electrical resistance, not ionized impurity scattering.^55^ While several works have explored Nb incorporation into this system, none have explicitly addressed the concept of “grain boundary complexion,″ which is the central focus of our current research. In this work, we provide a detailed examination of how Nb addition modifies the grain boundary without being incorporated into the crystal structure. Specifically, we demonstrate that in Bi-rich Mg_3_Sb_2_, the primary effect of Nb is to significantly reduce the grain boundary resistance, even when the grain size remains largely unchanged (Figure 1). This reduction in grain boundary resistance leads to a substantial improvement in *zT*, achieving a value of 1.14 at 330 K for Mg_3.1_Nb_0.1_(Sb_0.3_Bi_0.7_)_1.996_Te_0.004_, with a high weighted mobility of 335 cm^2^ V^–1^ K^–1^ and a low lattice thermal conductivity of 0.58 W m^–1^ K^–1^. For this sample, the device *ZT* is calculated to be 1.03 at 350 K, which increases to 1.24 at 523 K, and achieves a η_*max*_ of 10.5% at 623 K, highlighting its competitive performance.

## 2 Experimental Section

### 2.1 Sample Preparation

Samples of Mg_3.2–*x*_Nb_*x*_(Sb_0.3_Bi_0.7_)_1.996_Te_0.004_ (*x* = 0, 0.01, 0,025, 0.05, 0.1, and 0.15) were synthesized by mechanical alloying using a high energy ball mill (SPEX 8000D Mixer/Mill, 1425 rpm). All sample handlings during the synthesis and sintering processes were carried out in an Ar-filled glovebox to avoid oxidation. All the high-purity raw materials of magnesium powder (Mg, 99.8%; Alfa Aesar), bismuth pieces (Bi, 99.999%; Alfa Aesar), antimony shots (Sb, 99.999%; Alfa Aesar), tellurium pieces (Te, 99.9999%; Alfa Aesar), and niobium powder (99.98%, Alfa Aesar) were used without further purification. The stoichiometric amounts of the elements were loaded into a stainless-steel vial with two half-inch stainless steel balls (Figure 1). The mechanical alloying was applied for a duration of 2 h. Following that the resultant powders were loaded into a graphite die and compressed via cold press. For consolidation, spark plasma sintering (SPS) was applied under a uniaxial pressure of 50 MPa for 2 min at 1073 K under vacuum.

### 2.2 Sample Characterization

The crystal structure and phase purity analyses were carried out with room temperature X-ray diffraction (XRD, Rigaku Mini Flex 600, Cu K_α_ (λ= 1.5406 Å), 40 kV voltage and 15 mA current). The lattice parameters were refined, and texture analysis was performed using the WinCSD program package.^56^ The morphology and elemental compositions of the samples were determined by Scanning Electron Microscopy (SEM) and Energy Dispersive X-ray Spectroscopy (EDS), respectively, using a Zeiss Ultra Plus Field Emission Scanning Electron Microscope. Accurate chemical composition analyses were conducted with Wavelength Dispersive X-ray Spectroscopy (WDS) on an electron microprobe (Cameca SX 100, tungsten cathode) considering the line compound Mg_2_Si and certificated elements of Sb, Bi and Te as references. The high-resolution transmission electron microscopy (HR-TEM) and selected area electron diffraction (SAED) were employed on samples using the Talos F200S scanning/transmission electron microscope (S/TEM) operating with a maximum acceleration voltage of 200 kV.

### 2.3 Transport Property Measurements

The transport properties of samples were analyzed between 330 and 623 K. The thermal diffusivity, *D*, of the samples, was measured by Netzsch LFA 467 equipment on disc-shaped geometries (10 mm in diameter). The thermal conductivity, κ, values were then calculated by the relation, κ = *D* × *c*_*p*_ × *d*, where *c*_*p*_ is the specific heat estimated by polynomial equation described by Agne et al.^57^ (Figure S1), and *d* is the geometrical density. The *S* and σ measurements were conducted by Ulvac ZEM-3 device on bar-shaped samples. To achieve the desired geometry, samples were cut with a diamond wire saw. All the transport property measurements were performed along the cross-plane direction, which is parallel to the SPS pressing direction. The average *zT* values (*zT*_*ave*_) of the samples were determined using the formula *zT*_*ave*_ = ∫_*T*_*c*__^*T*_*h*_^*zT dT*/(*T*_*h*_ – *T*_*c*_), where *T*_*h*_ and *T*_*c*_ represent the hot-side and cold-side temperatures, respectively. The thermoelectric device figure of merit, *ZT*, and the maximum conversion efficiency, η_*max*_, were calculated according to the method described by Snyder et al.^11^

### 2.4 Effective Mass (EM) Model

Carrier concentration (*n*_*H*_)-dependent *PF* (=*S*^2^σ) and *zT* near room temperature were calculated for *x* = 0 and 0.05 in Mg_3.2–*x*_Nb_*x*_(Sb_0.3_Bi_0.7_)_1.996_Te_0.004_ using the Effective Mass (EM) model, assuming acoustic phonon scattering as the dominant carrier scattering mechanism.^58^ The density-of-states effective mass (*m*_d_*) was first determined by fitting theoretical *S* (eq 1) and *n*_*H*_ (eq 2) to experimental data. The *k*_*B*_, *e*, η, *h*, and *F*_*j*_ are the Boltzmann constant, electric charge, reduced Fermi energy, Planck’s constant, and the Fermi integral of order *j* (eq 3), respectively:
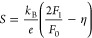
1
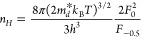
2
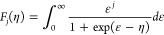
3

From the *m*_d_* and nondegenerate mobility (μ_*0*_, Table S3), the weighted mobility (μ_*W*_) was calculated using eq 4, where *m*_*e*_ is the electron rest mass. Using the μ_*W*_, the *n*_*H*_-dependent *PF* was predicted.
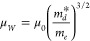
4

The thermoelectric quality factor (*B*-factor) was calculated using μ_*W*_ and the lattice thermal conductivity (κ_*l*_).
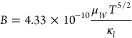
5

The *B*-factor was used to predict the *n*_*H*_-dependent *zT*.

### 2.5 Thermal Conductivity model

The apparent κ_*l*_ (eq 5) near room temperature was obtained by subtracting electronic contribution to κ (κ_*e*_) from the experimental κ (κ_*l*_ = κ – κ_*e*_). The κ_*e*_ was, in turn, calculated from Lorenz number (*L*) and σ (eq 6).
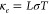
6

## 3 Results and Discussion

### 3.1 Phase and Crystal Structure Analysis

**Figure 2:**
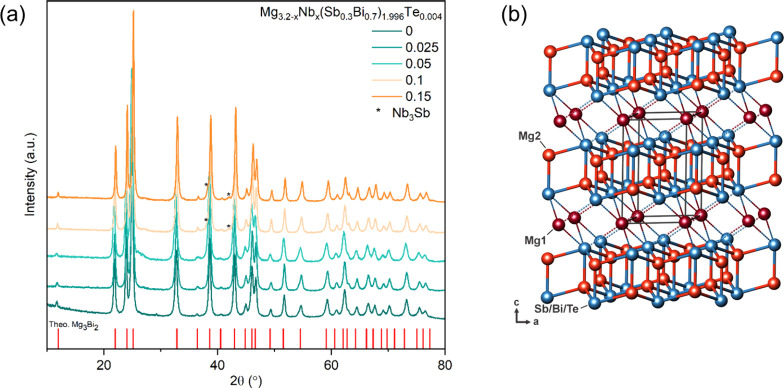
a) XRD patterns (Cu–K_∝_ radiation) of Mg_3.2–*x*_Nb_*x*_(Sb_0.3_Bi_0.7_)_1.996_Te_0.004_ (*x* = 0, 0.025, 0.05, 0.1 and 0.15), b) the crystal structure of Mg_3_(Sb,Bi)_2_ with possible atom positions.

The XRD patterns for pristine and Nb-added samples of Mg_3.2–*x*_Nb_*x*_(Sb_0.3_Bi_0.7_)_1.996_Te_0.004_ (*x* = 0, 0.025, 0.05, 0.1 and 0.15) are given in Figure 2a. The XRD pattern of the pristine sample conforms to the trigonal Mg_3_Bi_2_ crystal structure (space group: *P*3̅*m*1, Figure 2b). However, the Sb^3–^ substitution on Bi^3–^ sites shifts peak positions toward higher 2θ values because of the smaller ionic radius of Sb^3–^ (1.88 Å) compared to Bi^3–^ (1.93 Å). On the other hand, no detectable shift in peak positions is observed for the Nb-added samples due to similar ionic radii of Mg^2+^ and Nb cations (See Table S1 and Figure S2). A shoulder peak and a low-intensity secondary peak emerged at around 38° and 42° for the samples with *x* = 0.1 and 0.15, indicating the presence of the cubic Nb_3_Sb phase (space group: *Pm*3̅*n*). This shows that the solubility of Nb in the target phase is significantly lower than the added amounts. This is consistent with the results from Luo et al.^33^ for Mg_3_Sb_2_ that showed only metallic Nb with no detectable incorporation into the target phase.

### 3.2 Microstructure and HR-TEM Analysis

**Figure 3:**
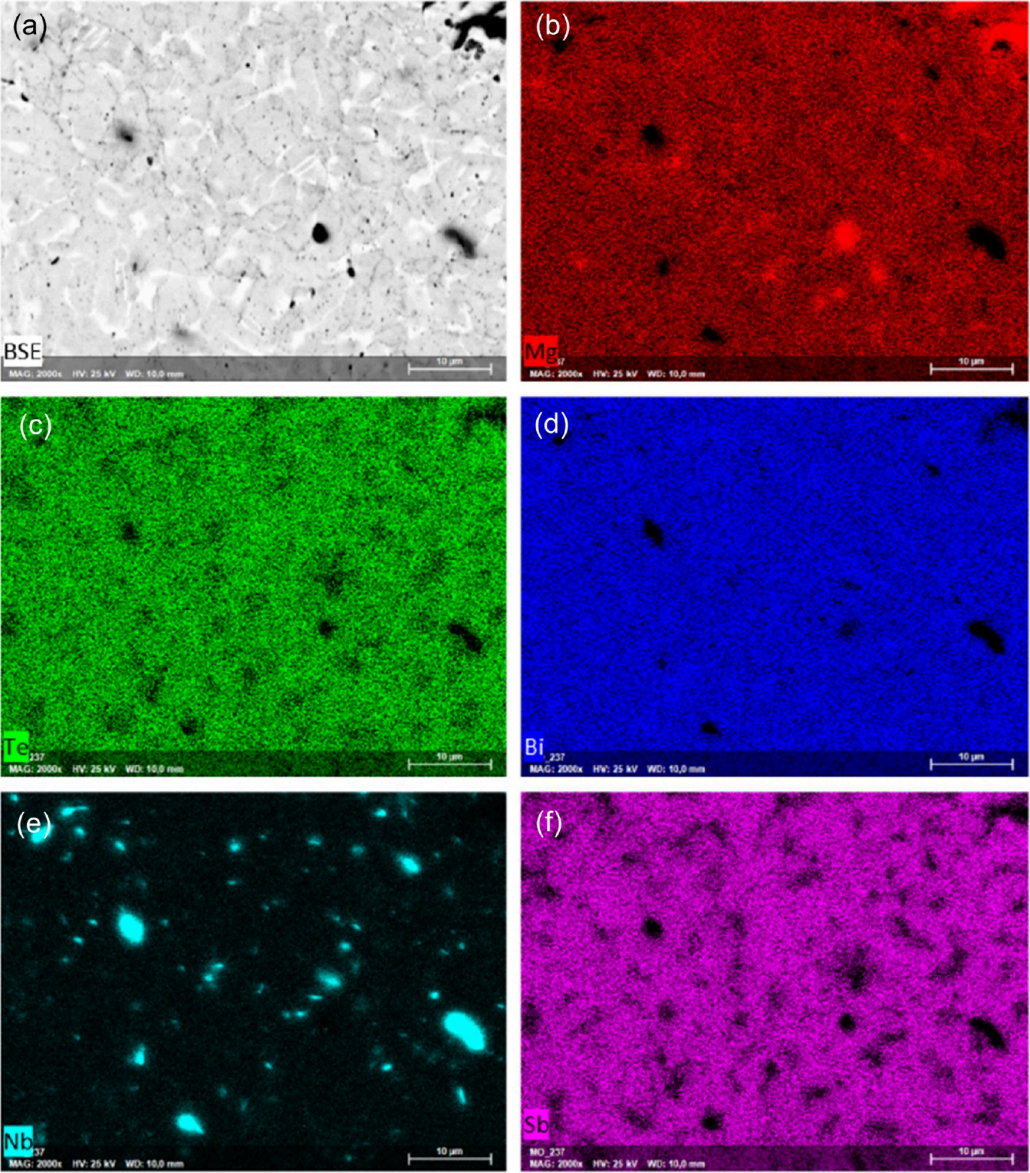
EDS elemental mapping of the Mg_3.1_Nb_0.1_(Sb_0.3_Bi_0.7_)_1.996_Te_0.004_ sample.

The EDS mapping of the Mg_3.1_Nb_0.1_(Sb_0.3_Bi_0.7_)_1.996_Te_0.004_ sample is shown in Figure 3. SEM images reveal a homogeneous elemental distribution of Sb, Bi, and Te, with black spots corresponding to the lighter elements of Mg and Nb. The elemental secondary phases are predominantly observed around grain boundaries but within the grains as well. The detection of Te was not possible because of its low concentration. Elemental mapping shows that a significant portion of Nb remains in its elemental form rather than incorporating into the crystal structure. Furthermore, distinct phase differences are discernible in the microstructure, highlighted by variations in contrast. EDS analysis elucidates the elemental distribution: the darker gray areas correspond to 59.9, 25.8, 12.0, and 2.4 at. % of Mg, Bi, Sb, and Nb, respectively. In contrast, brighter areas exhibit concentrations of 59.0, 28.4, 10.6, and 2 at. % of Mg, Bi, Sb, and Nb. Thus, a meaningful difference is observed in the amounts of Bi for the bright and gray areas.

**Figure 4:**
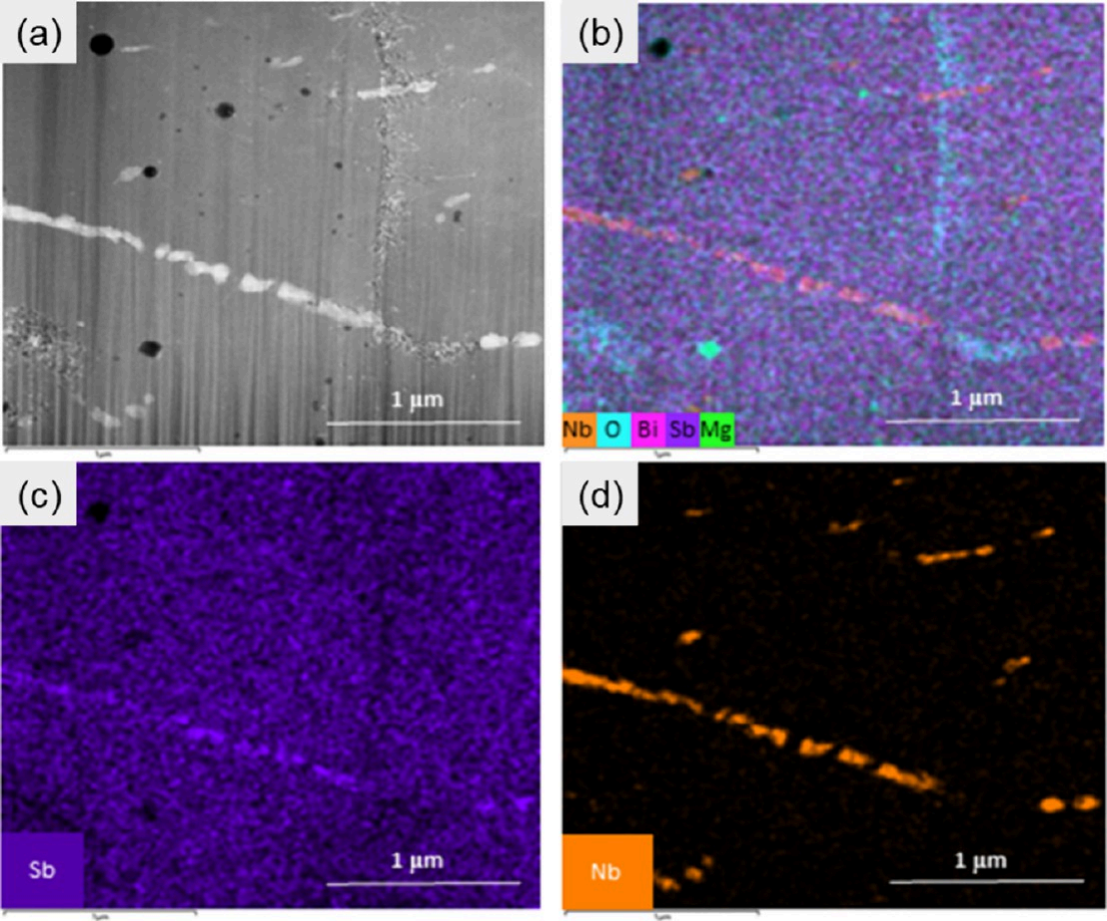
a) STEM image of Mg_3.05_Nb_0.15_(Sb_0.3_Bi_0.7_)_1.996_Te_0.004_, b) EDS overall elemental mapping, c) elemental mapping of Sb L_α1_ and d) Nb K_α1_.

As discussed before, with the increase in Nb addition, the formation of the Nb_3_Sb secondary phase was observed from the XRD analysis. For further investigation of secondary phases and microstructure, a STEM image was captured from Mg_3.05_Nb_0.15_(Sb_0.3_Bi_0.7_)_1.996_Te_0.004_ sample, to highlight changes more prominently. In Figure 4, the STEM image, along with EDS layered images, elucidates the generation of the Nb_3_Sb phase within the microstructure. While the secondary phase of elemental Nb remained as small-grain precipitates (approximately 100 nm in size), the Nb_3_Sb phase manifested as distinct lines within the microstructure.

In addition to EDS analysis, WDS analysis was applied to both Nb-added and pristine samples. WDS images and elemental distribution graphs in Figure S3 further corroborate that darker spots correspond to the presence of Mg or Nb. From 10 different spots selected on the BSE image, the average chemical composition was calculated as Mg_3.13_Nb_0.09_Sb_0.6_Bi_1.37_Te_0.003_. Given that the spot size of the WDS measurement is approximately 5 μm and secondary phases of Nb are thoroughly precipitated in the microstructure, the incorporation of Nb into the crystal structure of the target phase remains ambiguous.

**Figure 5:**
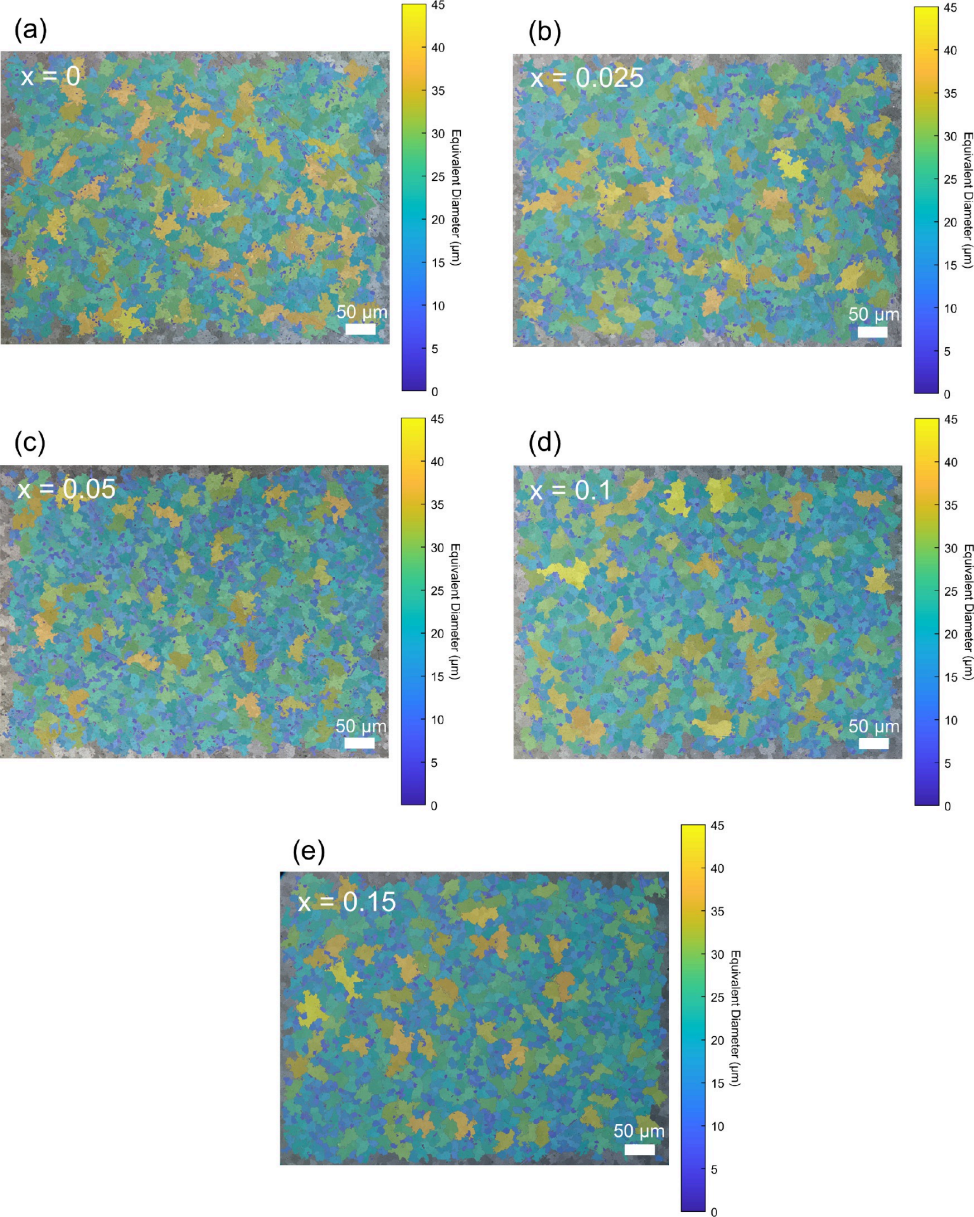
a–e) Grain size analysis of the pristine and Nb added Mg_3.2–*x*_Nb_*x*_(Sb_0.3_Bi_0.7_)_1.996_Te_0.004_ (*x* = 0, 0.025, 0.05, 0.1 and 0.15) samples.

Since clear estimations of grain boundaries and grains are challenging through SEM analysis, polarized light optical microscopy images were obtained both for the pristine and Nb-added samples. For quantitative average grain size analysis, an image analysis software (MIPAR) was utilized, generating the images provided in Figures 5a-e. The equivalent diameters of the grains were evaluated. The results unveiled essentially the same grain sizes, ranging from 13.97 to 16.52 μm, for pristine and Mg_3–*x*_Nb_*x*_(Sb_0.3_Bi_0.7_)_1.996_Te_0.004_ (*x* = 0, 0.025, 0.05, 0.1 and 0.15) samples (Table S2).

**Figure 6:**
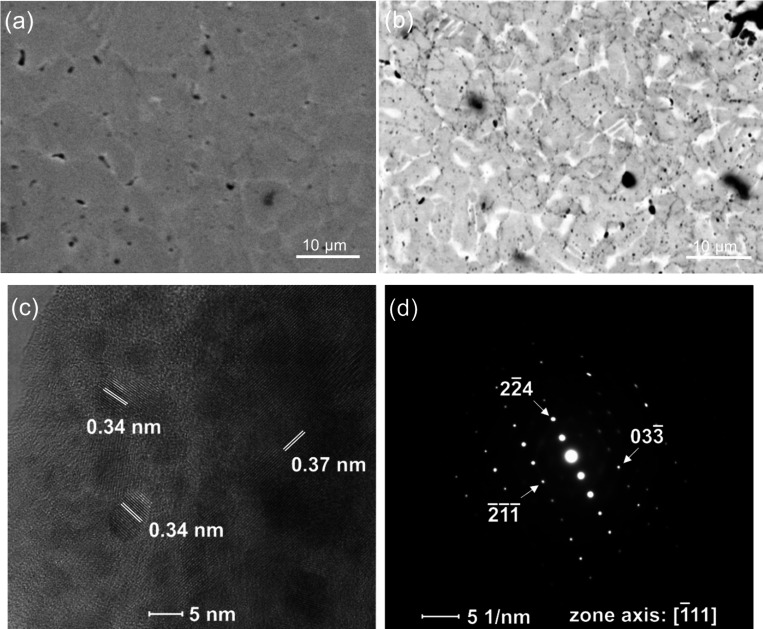
BSE-SEM images of Mg_3.2–*x*_Nb_*x*_(Sb_0.3_Bi_0.7_)_1.996_Te_0.004_ for a) *x* = 0 and b) *x* = 0.1. c) HR-TEM and d) SAED images for *x* = 0.1 sample.

Figure 6 presents the backscattered electron-scanning electron microscopy (BSE-SEM) images of both pristine and Nb-added sample of Mg_3.1_Nb_0.1_(Sb_0.3_Bi_0.7_)_1.996_Te_0.004_. In Figure 6a, the darker points observed correspond to the elemental Mg. The regions with different contrasts in BSE-SEM images reveal at least three distinct phases in both samples: a slightly Bi-excess phase, a slightly Bi-deficient phase, and secondary impurity phase manifesting as small black spots. EDS analysis indicates that the brighter areas creating inner grain boundaries possess a higher Bi content compared to the main grains with a gray color (Figure 3). This observation aligns with the study conducted by Sepheri-Amin et al., discussing the brighter contrast of grain boundaries due to a higher concentration of heavier elements in Mg_3_Sb_2_–Mg_3_Bi_2_ alloys.^26^ According to the EDS analysis, the overall composition of the pristine sample is Mg_3.13_Sb_0.64_Bi_1.43_ and the Nb-added sample is Mg_3.09_Nb_0.098_Sb_0.62_Bi_1.39_, which is comparable to the WDS analysis (Figure 3 and S3).

The HR-TEM image of a sample with *x* = 0.1 revealed lattice fringes with spacings of approximately 0.34 and 0.37 nm, corresponding to the (011) planes of Bi-deficient and (002) planes of Bi-excess phases within Mg_3_(Sb, Bi)_2_ (Figure 6c). This result indicates that in addition to micron-sized grains, there are nanometer-sized subgrains of the target phase present in the microstructure, suggesting the formation of a mesoscale architecture. Moreover, the SAED analysis of this sample displayed a diffraction pattern with well-defined spots along the [-111] zone axis, consistent with the trigonal crystal structure of Mg_3_(Sb, Bi)_2_ (Figure 6d).

### 3.3 Electronic Transport

**Figure 7:**
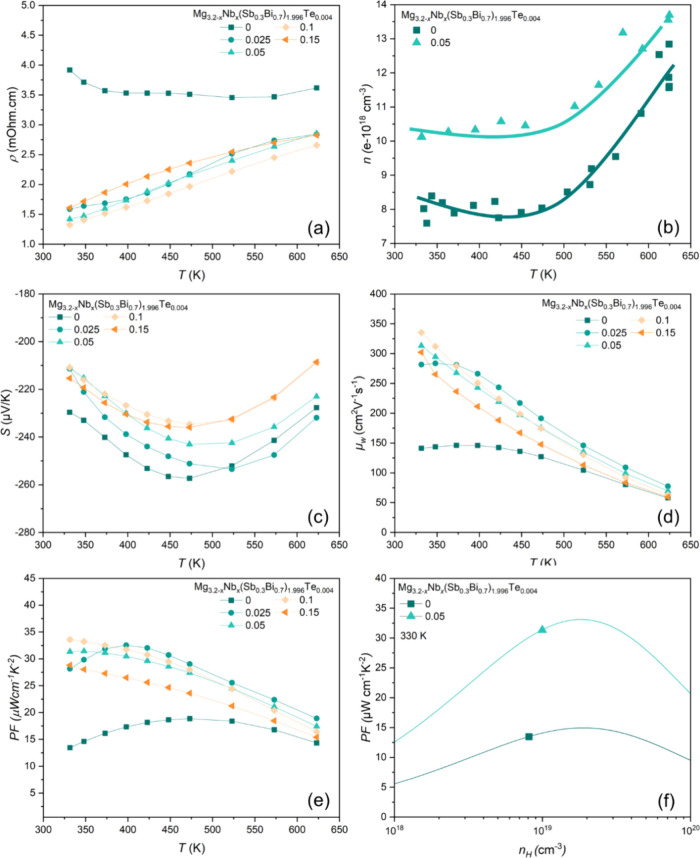
a) Electrical resistivity, b) *n_H_* (pristine and Nb added (*x* = 0.05) samples), c) *S*, d) *μ_W_*, e) *PF*, and f) estimated *PF* (lines) and measured *PF* (symbols) of pristine and Nb added Mg_3.2–*x*_Nb_*x*_(Sb_0.3_Bi_0.7_)_1.996_Te_0.004_ (*x* = 0, 0.025, 0.05, 0.1 and 0.1 5) samples.

From Figure 7a, it is evident that except for the pristine sample, the electrical resistivity (ρ = 1/σ) values of all Nb incorporated samples show a rising trend as the temperature increases in parallel with metal-like behavior. The sample without Nb exhibits a wide U-shaped temperature dependence of ρ due to competition between various carrier scattering mechanisms. At low temperatures, a thermally activated scattering dominates. While this is often attributed to ionized impurity scattering,^36^ it has been shown conclusively in Mg_3_Sb_2_ to be due to charged grain boundaries.^46^ Also, as shown in Figure S6, due to the scattering attributed to charged grain boundary, the sample without Nb exhibits U-shaped temperature dependence of Hall mobility (μ_*H*_), a phenomenon reported in various literature.^41,59−61^ The samples with and without Nb exhibit very similar temperature-dependent Seebeck coefficients (*S*), as shown in Figure 7c. This similarity suggests comparable doping levels and electron scattering mechanisms within the grains, along with a temperature-dependent weighted mobility (μ_*W*_). The calculation of μ_*W*_, band parameter directly proportional to theoretically achievable maximum *PF*, is utilized to understand influence of complex band behavior on transport properties. At low temperatures, μ_*W*_ increases and then transitions to the typical decreasing trend at higher temperatures, which is more pronounced in the *x* = 0 and 0.025 samples, as expected due to phonon scattering (Figure 7d).^40^ Since the μ_*W*_ incorporates the density of states, it is a more robust measure for materials with complex scattering mechanisms like grain boundaries or ionized impurities, which might not be fully captured by Hall effect measurement.^62^

The detection of secondary phases, such as Nb_3_Sb, through XRD (Figure 2a) and STEM images (Figure 4), does not seem to adversely impact ρ values either. In fact, Nb_3_Sb, known to exhibit superconductivity at very low temperatures, has a high σ with a room temperature resistivity of only 18 μΩ cm, suggesting a positive influence on the overall σ behavior.^63,64^ Similarly, the presence of Nb as another secondary phase, characterized by high σ, further supports the notion that these secondary phases might not have a detrimental effect on resistivity values. Moreover, the decrease in Sb content within the crystal structure due to secondary phase formation could play a role. Given the electron acceptor role of Sb atoms, a reduction in their quantity could lead to an increase in electron carrier concentration.

The carrier concentration (*n*_*H*_) measurements were conducted on both pristine and Nb-added (*x* = 0.05) samples. As shown in Figure 7b, the *n*_*H*_ exhibited only a slight increase following Nb addition. This observation is consistent with the slight reduction in *S* (Figure 7c). The decrease in ρ with Nb doping cannot be solely attributed to the increased *n*_*H*_. The μ_*H*_ of the *x* = 0.05 sample (440 cm^2^ V^–1^ s^–1^) is significantly higher than that of the pristine sample (200 cm^2^ V^–1^ s^–1^) (Figure S4), suggesting an additional factor influencing σ.

The change in σ is primarily attributed to the nature of the grain boundary complexions and a concurrent reduction in the influence of grain boundary scattering. It is well-established that grain boundaries have an important impact on electrical properties, particularly in Mg_3_Sb_2_-based thermoelectric materials. This has been shown by observing how the properties change with grain size from BSE-SEM images. Numerous studies have substantiated the impact of grain boundary resistance phenomena, particularly their contribution to elevated ρ values, especially at room temperature.^17,22,24,38−42^ The presence of Mg deficiencies around grain boundaries adds another layer to the explanation for the observed higher resistivity values.^42^

However, in this case, the reduction in grain boundary resistance is attributed not to grain growth but to a reduction in the resistance of the average grain boundary. This can be deduced from the polarized light images in Figure 5, illustrating no significant change in grain size upon Nb incorporation. Since there is no strong observable change in grain size to correlate with the reduction in grain boundary scattering upon Nb addition, Nb must be making the boundaries less resistive.

The magnitude of grain boundary scattering is determined by the space charge region that forms. This is possibly a result of a mismatch in conduction or valence band energies between the grain and grain boundary.^59,65^ For example, in a case where dopant segregation occurs or grain boundary wetting phases are formed, the band energies of the grain boundary region will change, and thus change the mismatch between the bulk and boundary conduction or valence bands. This will either reduce the barrier to conduction if the mismatch is lower or increase it if the mismatch is higher. Since Nb has been observed to form a grain boundary wetting phase in Mg_3_Sb_2,_^49^ this is a possible explanation for the reduction in grain boundary scattering observed between samples with and without Nb.

**Figure 8:**
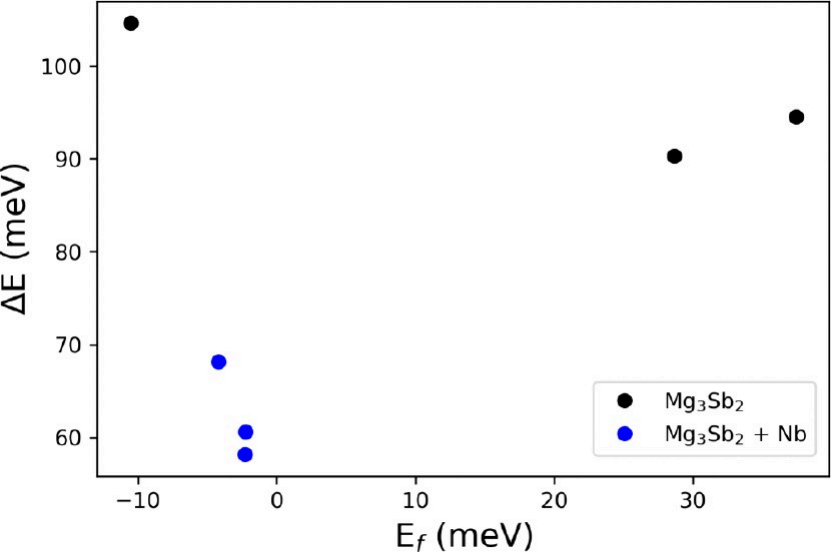
Modeled Δ*E* versus bulk E_f_ for Mg_3.2–*x*_Nb_*x*_(Sb_0.3_Bi_0.7_)_1.996_Te_0.004_ and one Mg_3.2_Bi_1.49_Sb_0.5_Te_0.01_ sample from Liu et al.^9^

This band energy mismatch between the boundaries and bulk, or band offset *ΔE*, can be modeled using a two-phase approach like that used by Kuo et al.^40^ In this case, a series circuit picture of grain boundary scattering can be used to separate grain and grain boundary contributions to conductivity:
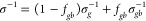
7where σ_*g*_ and σ_*gb*_ are the contributions to σ from grain and grain boundaries, respectively, and *f*_*gb*_ is the volume fraction of grain boundaries, which can be calculated from the grain size as described by Chookajorn et al.^66^ This equation can be coupled with the band transport equation for conductivity:
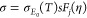
8where σ_*E*_0__is the transport coefficient, and *s* is a conduction mechanism-dependent parameter for the bulk (grain) and (*E*_*f*_ – Δ*E*)/*k*_*B*_*T* for the grain boundary phase. Phonon scattering parameters (*s* = 1 and σ_*E*_0__ ∝ *T*^0^) were used for both the grain and grain boundary phase. Single crystal σ values (*σ*_*g*_ in eq 7) for Mg_3.2_(Sb_0.3_Bi_0.7_)_1.996_Te_0.004_ can be calculated at different Fermi levels using eq 8, employing data from two samples with different grain sizes. This single crystal σ can be used to calculate a single crystal μ_*W*_, which is applicable to all samples with the same Bi–Sb ratio regardless of doping level until the onset of bipolar conduction.^62^ Using the calculated single crystal conductivity values (*σ*_*g*_), eq 7 is applied to determine the grain boundary conductivity (*σ*_*gb*_). The parameter *s* in eq 8 is treated as a constant, chosen by minimizing the root-mean-square error of eq 7 for Mg_3.2_(Sb_0.3_Bi_0.7_)_1.996_Te_0.004_ without added Nb. A series of temperature-dependent μ_*W*_ values can be calculated using the two-phase method with those derived from measured *S* and σ.^62^ The η value used to calculate the *σ*_*gb*_ can also be used to find the Δ*E* between the bulk and the grain boundaries. Figure 8 presents the results of this calculation, demonstrating that samples containing Nb exhibit a significantly lower Δ*E* compared to pristine samples. To show that this relationship is independent of grain size, we included data from Li et al.^51^ for a Mg_3.2_Bi_1.49_Sb_0.5_Te_0.01_ sample with a much smaller grain size of 2.9 μm and *E*_*f*_ of 37 meV. This sample exhibits a similar Δ*E* to the two samples synthesized in this study, both of which have larger grain sizes (>10 μm).

The model results indicate that the addition of Nb reduces Δ*E*, thereby decreasing the scattering strength of grain boundaries in Mg_3_Sb_2_. This effect is consistent across different grain sizes and doping levels, suggesting that the formation of the Nb wetting phase and resulting reduction in Δ*E* are the primary factors driving the decrease in grain boundary scattering with Nb addition.

Figure 7c demonstrates consistent *n*-type behavior across all samples, irrespective of Nb content. The decrease in *S* observed above 473 K is interpreted as compelling evidence of a bipolar effect.

The μ_*W*_ values of the samples were estimated from the experimental *S* and ρ data (Figure 7d).^62^ The μ_*W*_, which is independent of the chemical potential and *n_H_*, or the doping element, is an important band parameter for determining the electronic properties of thermoelectric materials.^4,26,62^ The onset of bipolar conduction observed in the *S* data above 473 K, attributed to a narrower band gap, leads to a decrease in the μ_*W*_ values.^26^ Consequently, comparisons of μ_*W*_ values below 473 K more accurately reflect the electronic band structures of the samples. Notably, after Nb metal addition, all μ_*W*_ values consistently increased, reaching a range of 280–330 cm^2^ V^–1^ s^–1^ at 330 K, regardless of the quantity of added Nb. In the temperature range of 330–450 K, the μ_*W*_ values exhibited a consistent increase with Nb addition (*x* = 0.025, 0.05, and 0.1), surpassing those of other promising low-temperature Mg_3_Sb_2_–Mg_3_Bi_2_ alloys where μ_*W*_ typically range between 180 and 250 cm^2^ V^–1^ s^–1^.^17^ This improvement stems from grain boundary complexions and the overall larger grain size compared to other studies. For example, at 330 K, the μ_*W*_ of the Nb-added sample with *x* = 0.1 (330 cm^2^ V^–1^ s^–1^) shows approximately 130% improvement over the pristine sample (142 cm^2^ V^–1^ s^–1^). Since the thermoelectric quality factor (*B*-factor in eq 5) is directly related to μ_*W*_ values,^67,68^ this substantial improvement also correlates with an enhancement in the theoretical maximum *zT*. The μ_*W*_ itself is also linked to the theoretical maximum *PF*. Experimentally, the *PF* of the Nb-added sample with *x* = 0.1 (33.5 μW cm^–1^ K^–2^) shows approximately 148% improvement compared to the pristine sample (13.5 μW cm^–1^ K^–2^) (Figure 7e). An even larger improvement, as predicted by the μ_*W*_ change, is possible if the *n*_*H*_ of the pristine sample is different from the optimum for the highest *PF*. Using the EM model, *n*_*H*_-dependent *PF* for the pristine and Nb-added sample with *x* = 0.05 are calculated (Figure 7f). As expected, the experimental *n*_*H*_ of the pristine sample (8 × 10^18^ cm^–3^) is lower than the optimum *n*_*H*_ for maximizing the *PF*, which is 2 × 10^19^ cm^–3^. We also observe that the *n*_*H*_ of the Nb-added sample with *x* = 0.05 is relatively closer to this optimal *n*_*H*_ (Figure S4 and Table S3).

### 3.4 Thermal Transport

**Figure 9:**
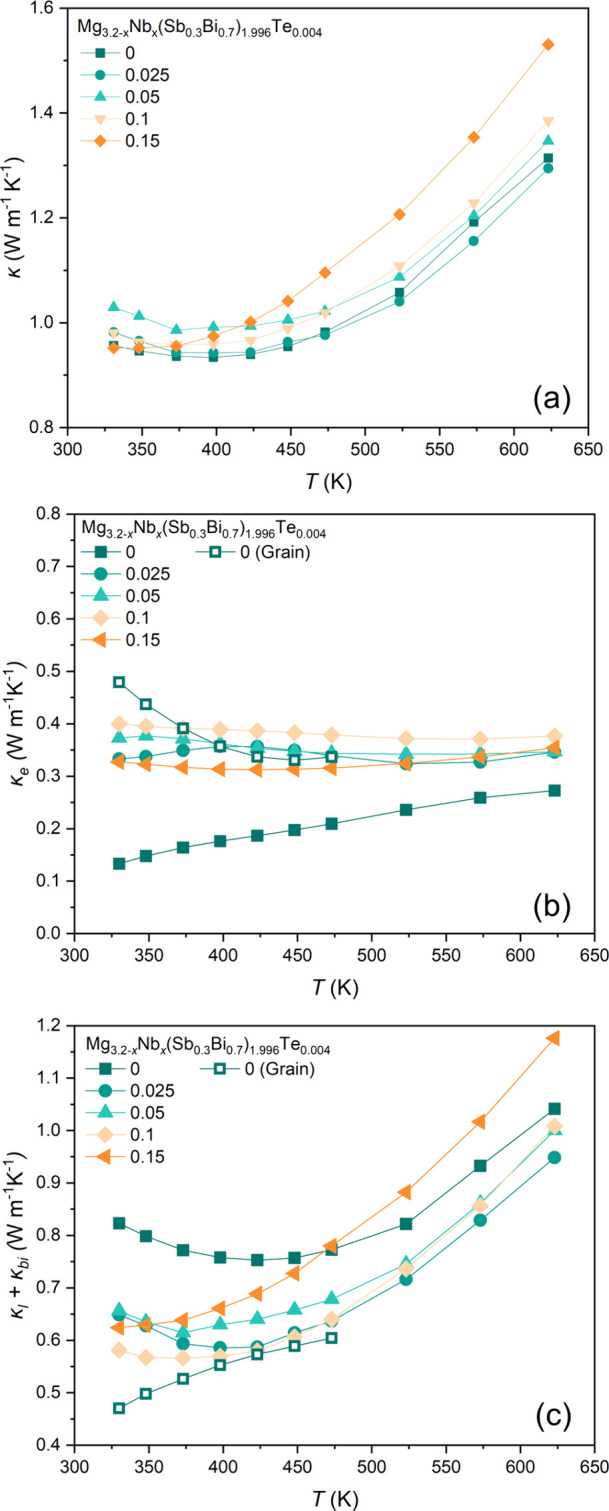
a) κ, b) κ_e_, and c) sum of κ_l_ and κ_bi_ of pristine and Nb-added Mg_3.2–*x*_Nb_*x*_(Sb_0.3_Bi_0.7_)_1.996_Te_0.004_ (*x* = 0, 0.025, 0.05, 0.1 and 0.1 5) samples.

Figure 9a presents experimental κ as a function of temperature. Both pristine and Nb-added samples exhibit a rising trend in κ with increasing temperature. Especially the pronounced increase in κ observed above 450 K coincides with the onset of the bipolar conduction demonstrated by the temperature-dependent *S* in Figure 7c. The EM model was used to estimate the electronic contribution (κ_*e*_) to κ for pristine and Nb-added samples (Figure 9b). The apparent lower κ_*e*_ of the pristine sample compared to the Nb-added samples near room temperature is attributed to the low σ_*gb*_ of the pristine sample, as demonstrated by Kuo et al.^69^ Given that the *S* is essentially the same for all samples (Figure 7c), it can be inferred that the σ_*g*_ is similar, and consequently, the κ_*e*_ is also comparable across samples. Figure 9b also presents the κ_*e*_ estimated by using σ_*g*_ instead of experimental σ in eq 6 (empty symbols). The close agreement between these values supports our inference about the similarity of σ_*g*_ across samples. The κ_*e*_ (Figure 9b) was subtracted from the κ (Figure 9a), to obtain the sum of lattice thermal conductivity (κ_*l*_) and bipolar thermal conductivity (κ_*bi*_), as shown in Figure 9c. The sum of κ_*l*_ and κ_*bi*_ estimated using κ_*e*_ derived from σ_*g*_ is also presented (empty symbols). After accounting for grain boundary scattering, the apparent higher κ_*l*_ of the pristine sample (filled symbols) becomes comparable to other Nb-added samples (empty symbols). Hence, Nb and Nb_3_Sb phases (observed in *x* ≥ 0.1 samples) were ineffective in suppressing κ_*l*_.

### 3.5 Thermoelectric Figure of Merit

**Figure 10:**
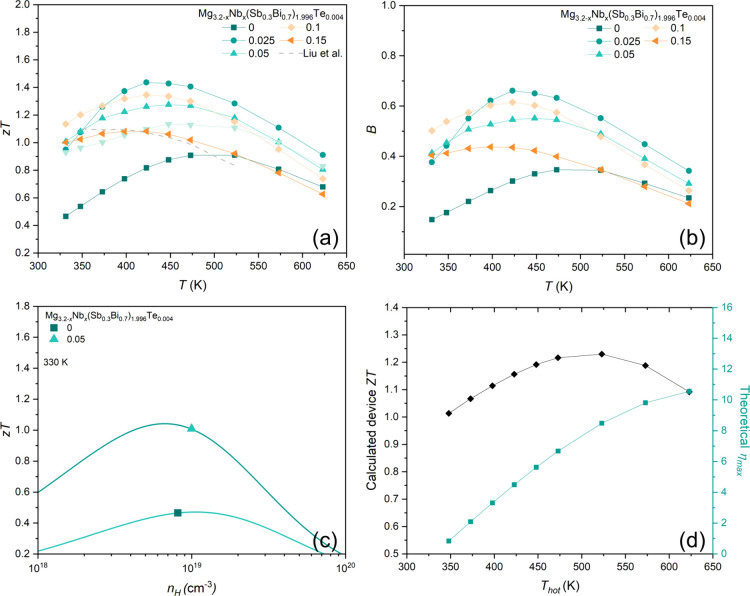
Temperature-dependent a) *zT* (Liu et al.^17^) in dashed line) and b) B-factor of pristine and Nb-added Mg_3.2–*x*_Nb_*x*_(Sb_0.3_Bi_0.7_)_1.996_Te_0.004_ (*x* = 0, 0.025, 0.05, 0.1 and 0.15) samples. (c) Calculated and measured n_H_-dependent *zT* of pristine and *x* = 0.05 samples, and d) calculated device *ZT* and η_max_ of *x* = 0.1 sample.

Figure 10 presents the temperature-dependent *zT* and *B*-factor, along with the theoretically achievable *zT* as a function of *n*_*H*_. The combined effect of low κ_*l*_ and high μ_*W*_, achieved through reduced grain boundary scattering, results in a *zT* exceeding 1 for Nb-doped samples between 330 and 423 K, making them competitive with state-of-the-art Bi_2_Te_3_ alloys.

As shown in Figure 10a, the sample with *x* = 0.025 exhibits a peak *zT* of 1.43 at 450 K, the highest among all investigated compositions. Notably, the *zT* of the *x* = 0.1 sample reaches 1.14 near room temperature, surpassing all reported Mg_3_(Sb,Bi)_2_-based materials. For comparison, the high *zT* of Cu-doped Mg_3_(Sb,Bi)_2_ by Liu et al. (dashed line) is included. To ensure reproducibility, an additional sample with identical composition was prepared, demonstrating comparable *zT* (Figure S5). The *x* = 0.025 sample achieves an average *zT* of 1.22 between 330 and 573 K. Figure 10b confirms the correlation between Nb doping and increased *B*-factors (proportional to μ_*W*_/κ_*l*_ in eq 5), which reflect the theoretical maximum *zT*.

Figure 10c presents the calculated *n*_*H*_-dependent *zT* for pristine and Nb-doped (*x* = 0.05) samples. The lines represent the theoretically achievable *zT* calculated using the EM model. Notably, the *zT* of the *x* = 0.05 sample could potentially reach ∼1.05 at an *n*_*H*_ of 7 × 10^18^ cm^–3^. However, the measured *zT* already approaches 1.01 at 330 K, suggesting near-optimal *n*_*H*_ in *x* = 0.05 sample.

The calculated device *ZT* for Mg_3.1_Nb_0.1_(Sb_0.3_Bi_0.7_)_1.996_Te_0.004_ ranges from 1.03 to 1.24 within the temperature range of 350 to 623 K, as presented in Figure 8d.^70^ At 623 K, the theoretical maximum device efficiency (η_*max*_) reaches an impressive 10.5%, demonstrating highly competitive performance.

## 4 Conclusions

The Mg_3_(Sb,Bi)_2_ system has been significantly modified through the incorporation of Nb, reducing the grain boundary electrical resistance. While in Mg_3_Sb_2_ the primary effect of Nb is to facilitate grain growth, the samples prepared here show little change in grain size. Instead, the resistivity of the grain boundaries themselves is dramatically reduced. The reduction is so significant that the conductivity and mobility have little indication of grain boundary resistance. This study shows that the properties of the grain boundary complexion phase itself in Mg_3_(Sb,Bi)_2_ can be modified with chemical processing. The achievement of low thermal conductivity in the range of 0.9–1 W m^–1^ K^–1^, coupled with low resistivity values and elevated carrier mobility, has contributed to the attainment of high *zT* values, particularly in low-temperature regimes. Notably, our study has yielded a record-high *zT* of 1.14 at 330 K, with a device *ZT* of 1.03 at 350 K, reaching 1.24 at 523 K, and achieving a η_*max*_ of 10.5% at 623 K, highlighting its competitive performance. Our work encourages the exploration of Mg_3_(Sb,Bi)_2_ as an alternative thermoelectric material for room-temperature applications, challenging the dominance of traditional materials like Bi_2_Te_3_. The emphasis on microstructure engineering, specifically the chemistry of grain boundary complexion phases, emerges as a promising avenue for further enhancing the thermoelectric properties of these materials.

## Supporting Information Available

Comprehensive data and analyses related to heat capacity, XRD patterns and lattice parameters, SEM images and grain size analysis, effective mass analysis, thermoelectric transport properties, and Hall and weighted mobility (PDF)
