# Supplementary material for: Realizing Ultrahigh Near-Room-Temperature Thermoelectric Figure of Merit for N-Type Mg3(Sb,Bi)2 through Grain Boundary Complexion Engineering with Niobium
Source: ACS Appl Mater Interfaces. 2024 Sep 24;16(39):52501–14. doi: 10.1021/acsami.4c12046 (PMC11577319; doi:10.1021/acsami.4c12046)
Supplement: Supplementary file 1 — am4c12046_si_001.pdf [file am4c12046_si_001.pdf]

Supporting Information

Realizing Ultrahigh Near-Room-Temperature Thermoelectric Figure of Merit for N-type $Mg_3(Sb,Bi)_2$ through Grain Boundary Complexion Engineering with Niobium

Melis Ozen[a,b,c], Arda Baran Burcak[d], Duncan Zavanelli[e], Minsu Heo[f], Mujde Yahyaoglu[b], Yahya Oz[c], Ulrich Burkhardt[g], Hyun-Sik Kim[f], G. Jeffrey Snyder[e], and Umut Aydemir*[b,d]

[a] Graduate School of Sciences and Engineering, Koç University, Istanbul-34450, Türkiye

[b] Koç University Boron and Advanced Materials Application and Research Center, Istanbul-34450, Türkiye

[c] Turkish Aerospace, R&D and Technology Directorate, Ankara 06980, Türkiye

[d] Department of Chemistry, Koç University, Sariyer, Istanbul, 34450, Türkiye

[e] Department of Materials Science and Engineering, Northwestern University, Evanston, IL-60208, USA

[f] Department of Materials Science and Engineering, University of Seoul, Seoul 02504, South Korea

[g] Max-Planck-Institut für Chemische Physik fester Stoffe, Dresden-01187, Germany

* Corresponding author, Email: uaydemir@ku.edu.tr

In this study to calculate the thermal conductivity, a polynomial expression described by Agne et al.[1] (Eq.1), was used to estimate the heat capacity values for $Mg_{3.2-x}Nb_x(Sb_{0.3}Bi_{0.7})_{1.996}Te_{0.004}$ (x = 0, 0.025, 0,05, 0.1, 0.15). Maier-Kelly polynomial expression was used to estimate the heat capacity values for the range 330-623K:

$$c_p = \frac{3NR}{M_W}\left(1 + 1.3 \times 10^{-4}T - 4 \times 10^{3}T^{-2}\right) \quad \text{Eq. 1.}$$

Where $3NR$=129.7 J mol^{-1}K^{-1}, M_W is the molecular weight (kg mol^{-1}) and T is the temperature in K. Figure S1 compares the heat capacity values calculated by polynomial expression and Dulong-Petit law.

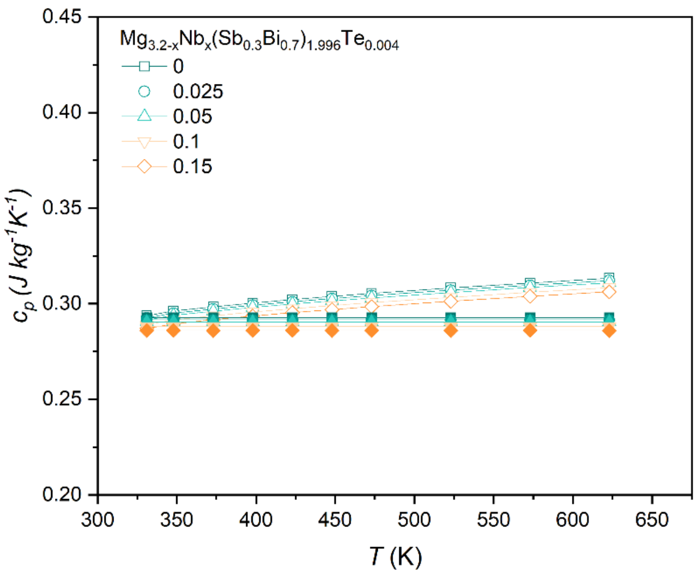

Figure S1. *Heat capacity values for $Mg_{3.2-x}Nb_x(Sb_{0.3}Bi_{0.7})_{1.996}Te_{0.004}$ (x = 0, 0.025, 0,05, 0.1, 0.15) where empty symbols are calculated by the polynomial Eq.1, and filled symbols are estimated by Dulong-Petit law.*

Table S1 tabulates the lattice parameters of $Mg_{3.2-x}Nb_x(Sb_{0.3}Bi_{0.7})_{1.996}Te_{0.004}$ (x = 0, 0.025, 0,05, 0.1, 0.15) samples determined using LaB_6 as a reference material. When the standard deviations are considered, no significant peak shift is observed for Nb-added samples.

Table S1. *Lattice parameters of $Mg_{3.2-x}Nb_x(Sb_{0.3}Bi_{0.7})_{1.996}Te_{0.004}$ (x = 0, 0.025, 0,05, 0.1, 0.15)*

With LaB_6 reference						Mg_3Bi_2[2]	Mg_3Sb_2[3]
	**0**	**0.025**	**0.05**	**0.1**	**0.15**		
***a* (Å)**	4.625(1)	4.6252(3)	4.6250(7)	4.6285(8)	4.6267(5)	4.666	4.5636(11)
***c* (Å)**	7.346(3)	7.3457(7)	7.350(2)	7.343(1)	7.351(1)	7.401	7.228(2)

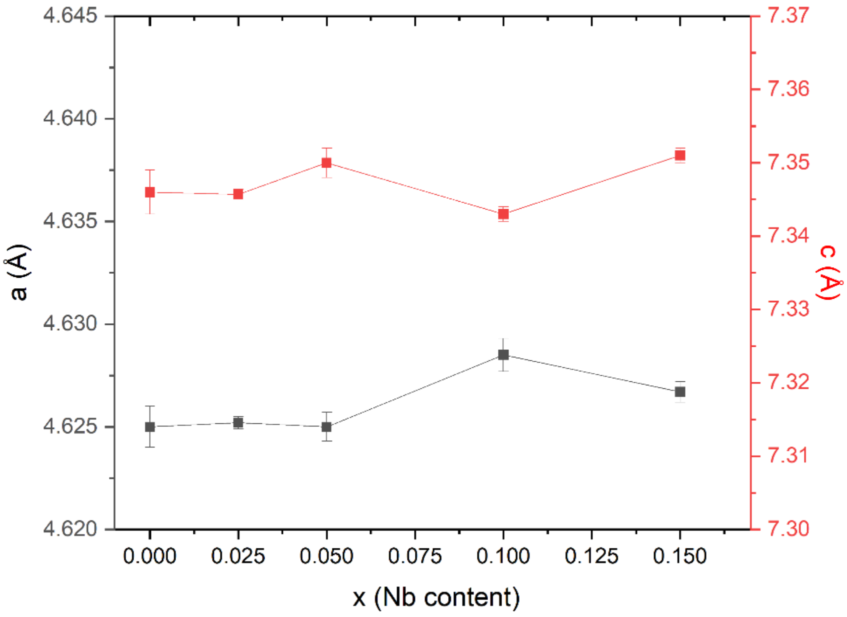

Figure S2. *Lattice parameters of $Mg_{3.2-x}Nb_x(Sb_{0.3}Bi_{0.7})_{1.996}Te_{0.004}$ (x = 0, 0.025, 0,05, 0.1, 0.15) measured with LaB_6 reference*

In Figure S3, BSE images of $Mg_{3.1}Nb_{0.1}(Sb_{0.3}Bi_{0.7})_{1.996}Te_{0.004}$ sample are presented. The red crosses mark the location where the EDS analysis was performed. Examining the results presented in Figure 3c and d (in the main text), it becomes evident that lighter areas correspond to the main phase, while darker regions are predominantly composed of elemental Nb. WDS analysis was conducted for ten different areas across the sample to ascertain the overall composition. Consequently, the average chemical composition was determined to be $Mg_{3.13}Nb_{0.09}Sb_{0.6}Bi_{1.37}Te_{0.003}$.

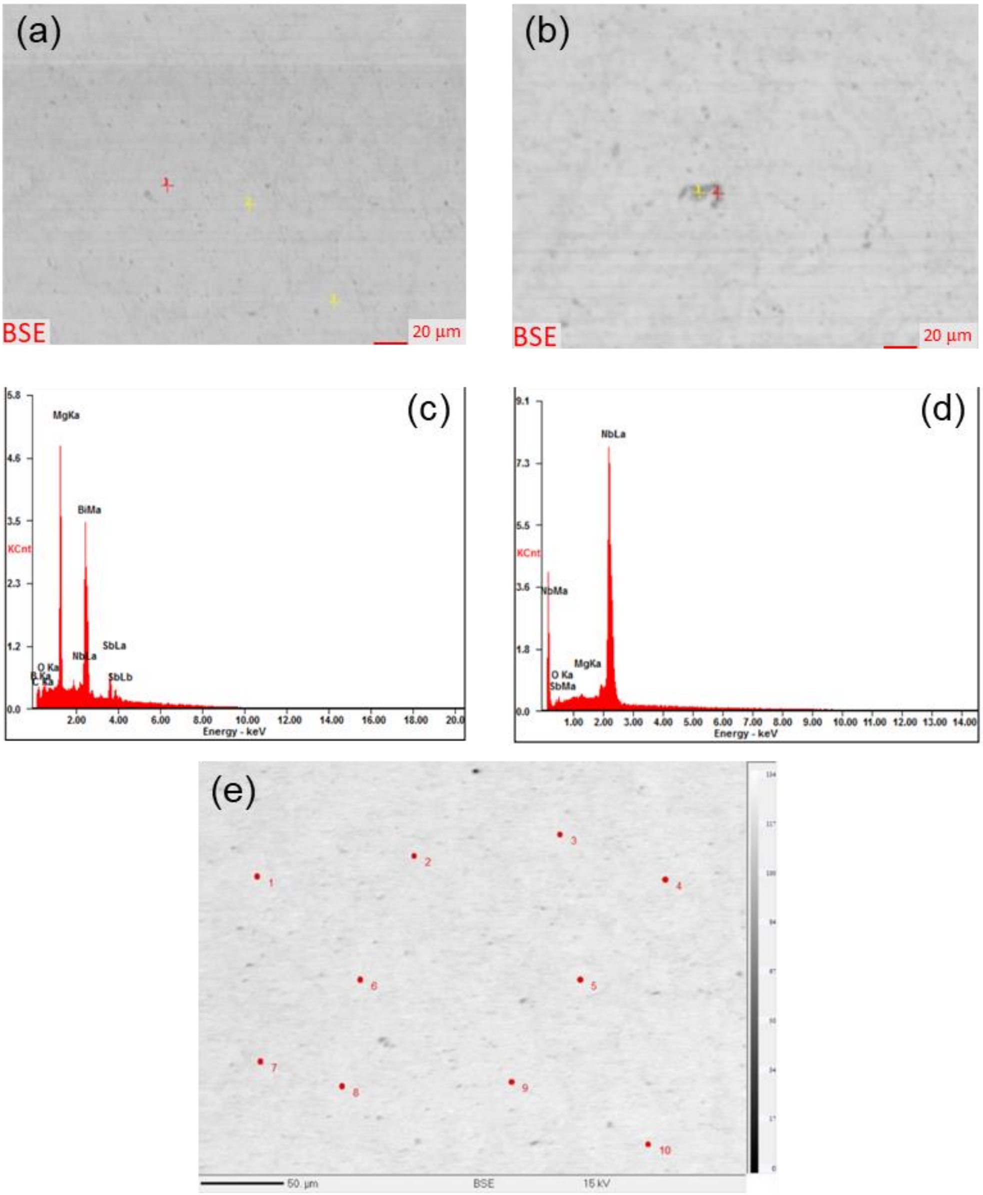

Figure S3. *a, b) BSE images, c,d) EDS analysis of* $Mg_{3.1}Nb_{0.1}(Sb_{0.3}Bi_{0.7})_{1.996}Te_{0.004}$ *from bright and dark areas, and e) ten different spots that WDS analysis was conducted on* $Mg_{3.1}Nb_{0.1}(Sb_{0.3}Bi_{0.7})_{1.996}Te_{0.004}$ *sample.*

Table S2: Mean equivalent diameters (grain sizes) of the samples.

Sample Composition	Mean Equivalent Diameter (μm)
$Mg_{3.2}(Sb_{0.3}Bi_{0.7})_{1.996}Te_{0.004}$	16.52
$Mg_{3.2}(Sb_{0.3}Bi_{0.7})_{1.996}Te_{0.004}$	13.97
$Mg_{3.175}Nb_{0.025}(Sb_{0.3}Bi_{0.7})_{1.996}Te_{0.004}$	14.63
$Mg_{3.15}Nb_{0.05}(Sb_{0.3}Bi_{0.7})_{1.996}Te_{0.004}$	13.51
$Mg_{3.1}Nb_{0.1}(Sb_{0.3}Bi_{0.7})_{1.996}Te_{0.004}$	15.21
$Mg_{3.05}Nb_{0.15}(Sb_{0.3}Bi_{0.7})_{1.996}Te_{0.004}$	15.40

Figure S4a and b show calculated (presented in lines) and experimental (presented in symbols) n_H-dependent S and Hall mobility (μ_H), respectively, of pristine and Nb-added $Mg_{3.15}Nb_{0.05}(Sb_{0.3}Bi_{0.7})_{1.996}Te_{0.004}$ (x = 0.05) at 330 K.

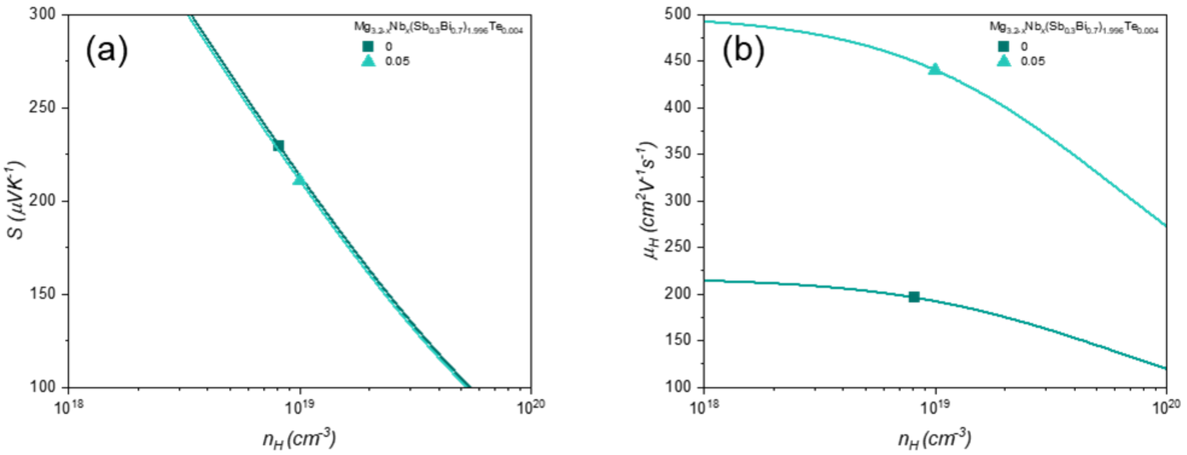

Figure S4. *Calculated (lines) and experimental (symbols) n_H-dependent a) S and b) μ_H of pristine and Nb-added $Mg_{3.15}Nb_{0.05}(Sb_{0.3}Bi_{0.7})_{1.996}Te_{0.004}$ (x = 0.05) samples at 330 K.*

Table S3 shows density-of-states effective mass (m_d^*), nondegenerated mobility (μ_0), and deformation potential (E_{def}) of pristine and Nb-added $Mg_{3.15}Nb_{0.05}(Sb_{0.3}Bi_{0.7})_{1.996}Te_{0.004}$ (x = 0.05) at 330 K.

Table S3. *Density-of-states effective mass (m_d^*), nondegenerate mobility (μ_0), and deformation potential (E_{def}) of pristine and Nb-added $Mg_{3.15}Nb_{0.05}(Sb_{0.3}Bi_{0.7})_{1.996}Te_{0.004}$ (x = 0.05) samples at 330 K.*

x	**m_d^* (m_e)**	**μ_0 (cm^2 V^{-1} s^{-1})**	**E_{def} (eV)**
0	0.694	246	6.3
0.05	0.678	564	4.3

The thermoelectric transport properties and *zT* values of Nb-added $Mg_{3.2}Nb_{0.1}(Sb_{0.3}Bi_{0.7})_{1.996}Te_{0.004}$ samples are presented in Figure S5. The *ρ* and *S* of the Nb-added sample with *x* = 0.1 from different batches show the same results according to ZEM-3 measurements. However, slight variations in *κ* values are observed, falling within the range of experimental error (typically around ± 5 %) for the LFA measurement technique. Combining both electronic and thermal transport properties, the calculated *zT* values are provided in Figure S5d. Both samples have high *zT* values near room temperature and mid-temperature ranges. At 330 K, the *zT* values of 1.08 and 1.14 were achieved, while the *zT* values reach 1.32 and 1.35 at 450 K for these two samples.

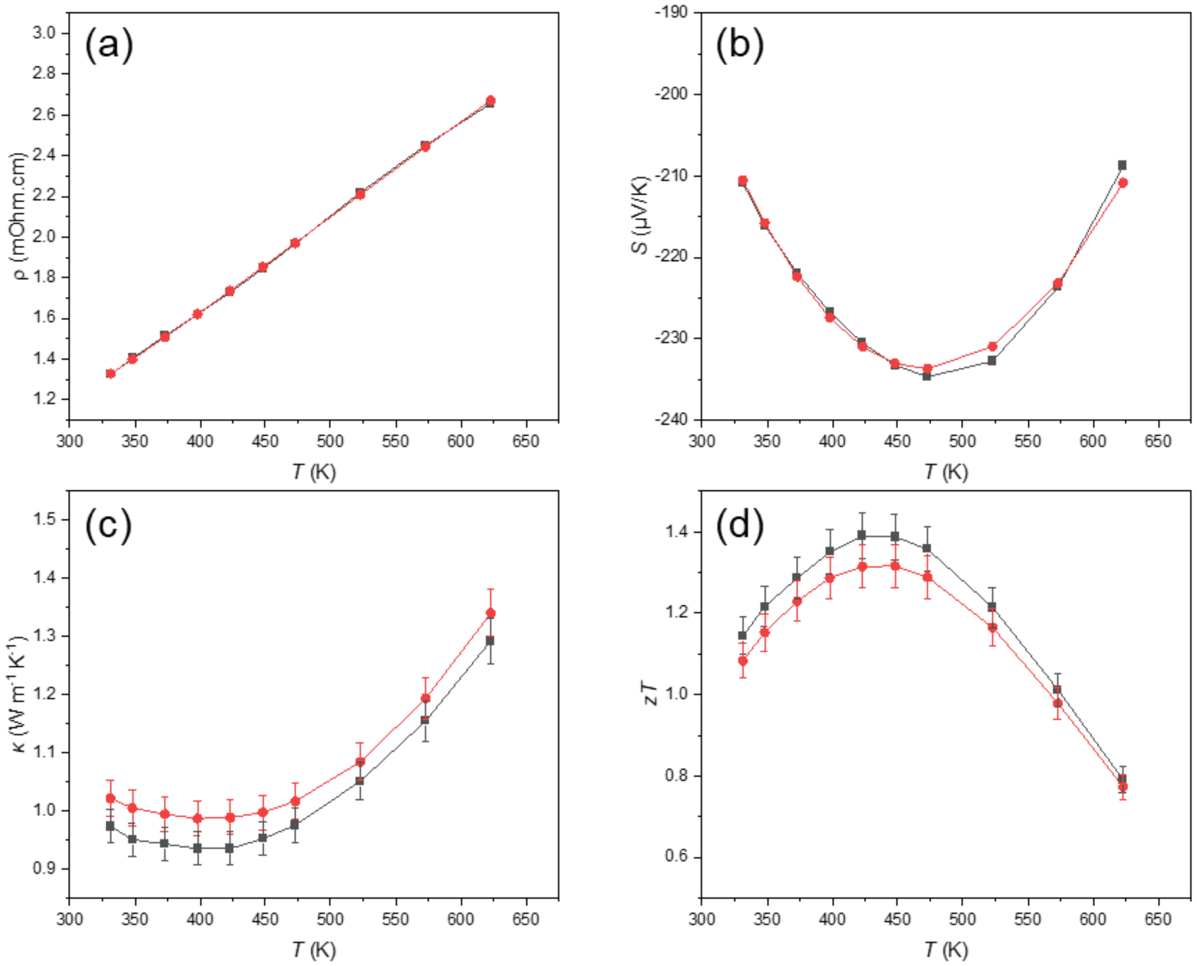

Figure S5. *Temperature-dependent a) ρ, b) S, c) κ, and d) zT values of two different* $Mg_{3.2}Nb_{0.1}(Sb_{0.3}Bi_{0.7})_{1.996}Te_{0.004}$ *samples.*

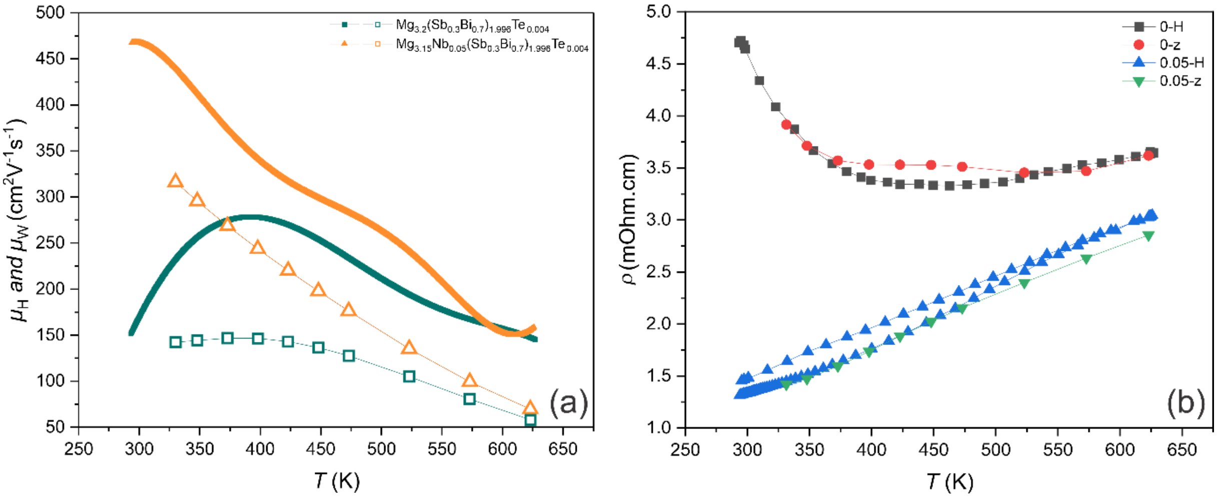

Figure S6. *a) Hall mobility (filled symbols) and weighted mobility (open symbols) of undoped and doped* $Mg_{3.15}Nb_x(Sb_{0.3}Bi_{0.7})_{1.996}Te_{0.004}$ *(x=0, 0.05) and b) resistivity values of undoped and doped* $Mg_{3.15}Nb_x(Sb_{0.3}Bi_{0.7})_{1.996}Te_{0.004}$ *(x=0, 0.05) (H= from Hall effect measurement, z= from ZEM3 measurement)*

References

1. Agne, Matthias T., et al. Materials Today Physics, 2018, **6,** 83-88.
2. E. Wigner, *Aufbau der Materie*, 1932, **19**, 203-216.
3. M. Calderón-Cueva, W. Peng, S. M. Clarke, J. Ding, B. L. Brugman, G. Levental, A. Balodhi, M. Rylko, O. Delaire and J. P. Walsh, *Chemistry of Materials*, 2021, **33**, 567-573.
